# Sensory and cortical biomarkers unveil pain modulation mechanisms induced by targeted multisensory neurostimulation

**DOI:** 10.1186/s12984-026-01998-5

**Published:** 2026-04-29

**Authors:** Giuseppe Valerio Aurucci, Noemi Gozzi, Andrea Cimolato, Markus Wagner, Carl Moritz Zipser, Stanisa Raspopovic

**Affiliations:** 1 Neuroengineering Laboratory, Department of Health Sciences and Technology, ETH Zürich, Tannenstrasse 1, 8092 Zürich, Switzerland; 2 Center for Medical Physics and Biomedical Engineering, Medical University of Vienna, 1090 Vienna, Austria; 3 Department of Neurology and Neurophysiology and Spinal Cord Injury Center, Balgrist University Hospital, University of Zurich, Zurich, Switzerland

**Keywords:** Pain, Neuromodulation, Neurostimulation, VR, Biomarkers, EEG, Neuropathy

## Abstract

**Background:**

Chronic neuropathic pain is a complex experience that poses a major challenge in personalized treatment. Identifying objective biomarkers of pain modulation is critical to validate emerging non-pharmacological therapies with reliable endpoints, overcoming the limitations of simplified subjective scales.

**Methods:**

Here, we introduce a multimodal monitoring framework that integrates behavioral, sensory, and cortical assessments to provide a comprehensive evaluation of a multisensory neurostimulation treatment combining immersive VR with targeted neurostimulation (VR+tSTIM). We compared the effects of this intervention with an active control in 18 participants with chronic neuropathic pain over multiple days.

**Results:**

VR+tSTIM led to a clinically significant reduction in self-reported pain intensity. This reduction was accompanied by sensory measures, with participants in the VR+tSTIM group showing enhanced tactile acuity and improved proprioceptive accuracy, effects that did not appear in the control group. Treatment effectiveness was further associated with cortical EEG signatures of decreased gamma and delta power together with increased alpha power.

**Conclusions:**

These findings identify potential sensory and cortical biomarkers associated with analgesia and suggest that pain relief in neuropathy may involve the modulation of both peripheral and central mechanisms. This comprehensive assessment paradigm establishes a foundation for the objective monitoring of treatment efficacy and advances the search for mechanistic biomarkers of pain modulation in clinical neuroengineering.

*Trial registration*: This study was approved by the Kantonale Ethikkommission Zürich (Nr. 2021–02258).

**Supplementary Information:**

The online version contains supplementary material available at 10.1186/s12984-026-01998-5.

## Background

Neuropathic pain is a chronic condition with substantial societal and economic impact, affecting approximately 7–10% of the population [1, 2]. It arises from damage or dysfunction within the central or peripheral nervous system [3] and is a frequent consequence of conditions such as diabetes, infections, spinal cord injury, cancer, and stroke [1, 4]. Regardless of etiology, participants often experience common symptoms, including sensory components (burning, shooting, and uncomfortable sensations [5]) and subjective factors. Indeed, alongside the sensory bottom-up components, chronic pain accounts for top-down emotional and cognitive factors directly modulating the descending pain pathways [6, 7]. This complex and multifaceted interplay of different pain dimensions poses a significant challenge for the development of effective therapies.

As of today, non-specific pharmacological intervention [8], such as commonly prescribed opioid analgesics, remains the most widely adopted approach. Yet, they their efficacy [9] and long-term effects remain limited [10–12], and they often lead to strong side effects [13], addiction and overdose problems [14]. This has motivated an increasing shift toward non-pharmacological interventions capable of engaging multiple pain pathways in a safer and more personalized manner. Among such alternatives, peripheral non-invasive transcutaneous electrical nerve stimulation (TENS) has long been considered a promising method for eliciting peripheral analgesia. Neurostimulation induces peripheral analgesia by multifactorial processes [15], triggering the pain-gate mechanism proposed by Melzack and Wall [16]: the stimulation of large-diameter sensory fibers elicits inhibitory mechanisms, blocking the processing of pain-related information. Alongside neuromodulation, Virtual Reality (VR) has been employed to modulate pain perception [17, 18] through top-down mechanisms such as attentional distraction [19, 20] and reorganization of body representation [21, 22], which have a neural connection to the processing of noxious stimuli [23].

In our previous works, we showed that the use of targeted neurostimulation [24] and its combination with VR [25] can clinically meaningfully reduce neuropathic pain over repeated sessions. Yet, despite its clinical potential, demonstrating therapeutic engagement through reliable and objective clinical endpoints remains an unresolved challenge [26]. Indeed, current clinical standards still rely primarily on single-visit, one-dimensional scales, which are prone to bias and incapable of reflecting the complex and fluctuating nature of neuropathic pain [26, 27]. Recognizing these limitations, healthcare agencies and researchers now advocate for comprehensive, multidimensional assessments that integrate behavioral, sensory, and physiological indicators [26, 28, 29]. Such approaches are essential not only to validate treatment efficacy but also to understand how therapies modulate different facets of neuropathic pain. In this context, EEG represents a particularly valuable neurophysiological tool in pain research because it is noninvasive, widely accessible, relatively easy to acquire repeatedly, and well suited for longitudinal monitoring in clinical settings. At the same time, its millisecond level temporal resolution enables the investigation of rapid neuronal population dynamics and pain related oscillatory signatures across frequency bands, including alterations linked to thalamocortical dysrhythmia and to the broader pain connectome [30–33].

To this end, we designed a comprehensive monitoring paradigm to jointly assess behavioral, sensory, and neurophysiological changes and evaluate the effectiveness of our multisensory pain intervention combining immersive VR with targeted neurostimulation (tSTIM). We compared the tSTIM + VR intervention with an active comparator in 18 participants with chronic neuropathic pain over multiple days. This study is based on the same experimental protocol and patients as in [25]; however, while the previous short report was limited to clinical analgesic outcomes, the present work substantially focuses on unveiling the underlying mechanisms of the observed outcomes through a comprehensive and complementary analysis. We combined detailed neuropathic pain profiling with quantitative sensory and neurophysiological measures to capture treatment effects more broadly and to investigate objective markers of pain modulation over time. Within this framework, we evaluated changes in tactile acuity, proprioception, and cortical activity, and examined their relationship with subjective pain reports. This approach aims to validate targeted, non-pharmacological interventions through both clinical and objective endpoints, advancing the development of reliable biomarkers of pain modulation.

## Methods

### Participants recruitment

 Participants were recruited through the Department of Neurology and Neurophysiology at the Balgrist University Hospital, University of Zurich, Switzerland. The inclusion and exclusion criteria are presented in Table S1. 25 participants were preliminarily assessed for eligibility. Participants who did not meet all the inclusion criteria were discarded. A total of 18 participants participated in the study. All participants read and signed the informed consent form including the use of identifiable images and access to medical records. The experiments were designed and conducted in accordance with the Declaration of Helsinki and approved by the Kantonale Ethikkommission Zürich (Nr. 2021–02258).

### Multisensory platform

**Fig. 1:**
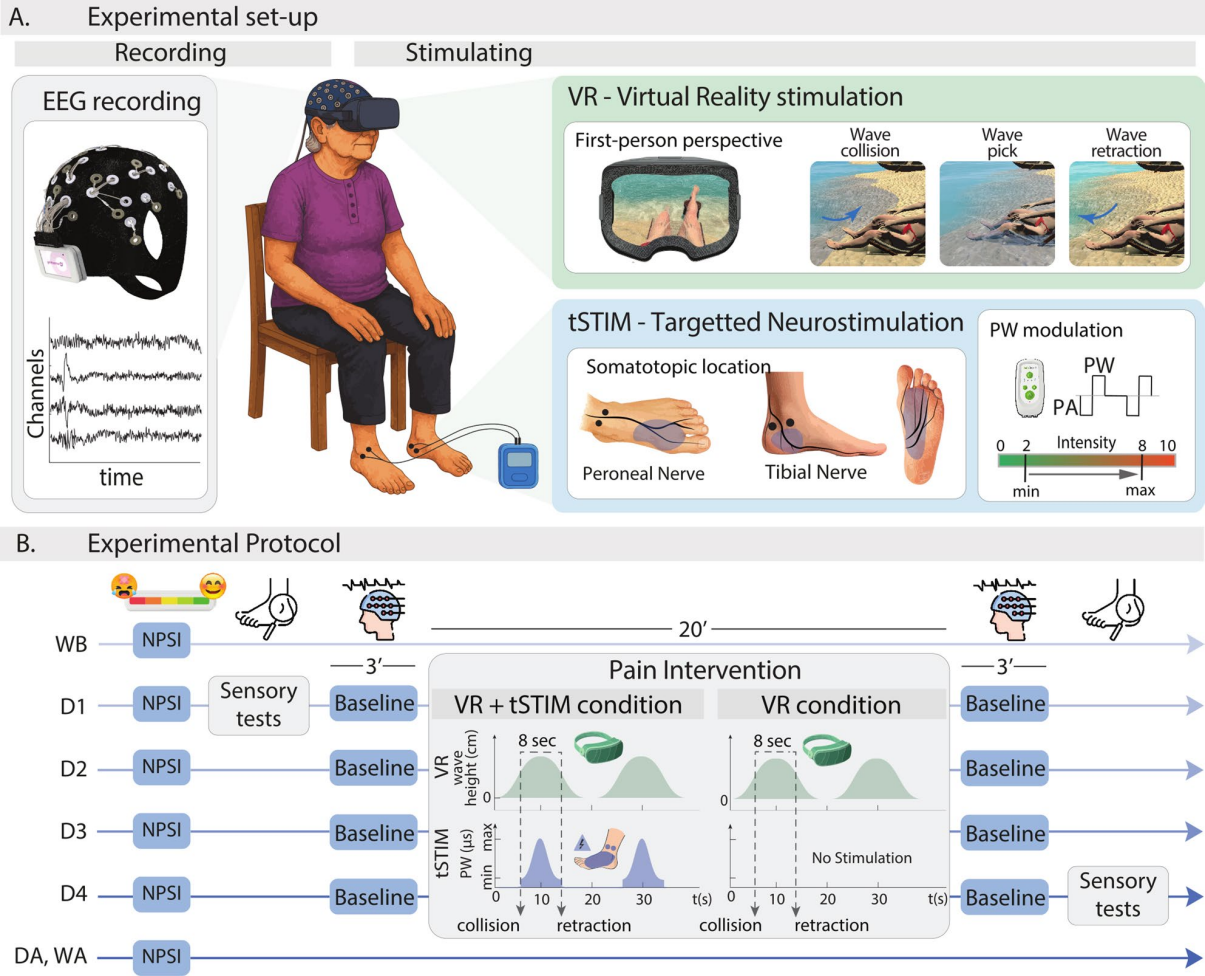
Multisensory pain-intervention protocol. A Multisensory experimental set-up. Participants received targeted neurostimulation (tSTIM) of the peroneal and tibial nerves while simultaneously being immersed in a first-person VR environment. The visual scene displayed a wave approaching and contacting the avatar’s foot, synchronized with the somatosensory sensation elicited by the electrical stimulation. The stimulation pulse width followed a Gaussian modulation, ranging from low intensity (2/10) to strong non painful intensity (8/10), matching the virtual wave’s height. Cortical activity was monitored with a 24 channel EEG system. B Experimental protocol. Participants completed twenty-minute intervention sessions across four consecutive days (D1 to D4). Baseline EEG was recorded before and after each session. Sensory assessments were performed on the first and last intervention days (D1 and D4). The Neuropathic Pain Symptom Inventory (NPSI) was collected at every session and at three additional moments: one week before the intervention (WB), the day after completion (DA), and one week later (WA). Participants were randomly assigned to either the VR+tSTIM condition, which provided synchronized visuo tactile stimulation, or the VR only condition, which delivered visual stimulation without tSTIM

We developed a multisensory platform (Fig. 1) to deliver non-invasive targeted neurostimulation real-time matched with the visual VR stimulus.

The technological framework combines a VR headset (HTC VIVE Pro) with a neurostimulation device (RehaMove3 Hasomed Gmbh) (Fig. 1A). The platform was designed similar to previous studies [25, 34–36]. To collect the neurophysiological signature of pain, a 24-channel portable EEG device (SMARTING MOBI, mBrain Train) with a sampling frequency of 500 Hz was used (Fig. 1A). The VR environment was developed in Unity 3D (version 2019.4) and consisted of a beach scenario in which participants saw themselves from a first-person perspective while sitting on a beach chair in front of the sea [25]. During the intervention, the participants viewed virtual waves repeatedly washing over their avatar’s feet (Fig. 1A). At the moment of wave–foot contact, targeted stimulation was delivered to the tibial and peroneal nerves of both legs, evoking touch-like sensations that spread naturally across the plantar and dorsal surfaces. To enhance multisensory congruence, the electrical stimulation was calibrated for each participant and modulated over time: the pulse width followed a Gaussian profile, starting at wave onset, peaking at the crest, and returning to baseline during retraction (Fig. 1A).

### Study design

Participants were randomly allocated to one of two groups using a computer-generated randomization sequence based on random permutation. In Group I (VR+tSTIM group), participants experienced a combined visuo-tactile VR and targeted neurostimulation. Group II (VR Group) received only the VR intervention without any electrical stimulation (Fig. 1B). The required sample size was determined using G*Power, based on an effect size of 1.58 for TENS intervention compared to sham TENS in the treatment of neuropathic pain [15]. An alpha error probability (α) of 0.05 and a statistical power of 0.8 were chosen. The calculation indicated a minimum of 8 participants per group. Except for the type of intervention provided, the experimental protocol was the same for both the groups (Fig. 1B). One week before (WB) the beginning of the intervention sessions, participants completed comprehensive pain questionnaires (Neuropathic Pain Symptom Inventory (NPSI) encompassing all the different neuropathic pain dimensions [37]. Participants received the pain intervention for four consecutive days (D1-4). NPSI were collected at the beginning of each session to assess the pain of the previous 24 h. The intervention consisted of 20 min of VR+tSTIM or VR, depending on the experimental group. An EEG baseline session of three minutes was recorded, before and after the pain intervention. To assess the impact of the intervention on sensory recovery and changes with respect to the severity of sensory deficits associated with neuropathy, participants underwent two standardized clinical tests, on the first and last intervention days: the Two-Point Discrimination (2PD) Test [38] and the Proprioceptive Displacement Test [39]. As a follow-up, participants also completed the Neuropathic Pain Symptom Inventory (NPSI) one day (DA) and one week (WA) after the intervention (Fig. 1B). This study reports an extended analysis of a previously published cohort and experimental protocol [25]. While the participant cohort overlaps, the analyses presented here focus on multidimensional pain assessment, sensory outcomes, and neurophysiological biomarkers that were not fully reported previously.

### tSTIM calibration

In the VR+tSTIM group, tSTIM calibration was performed at the beginning of each session, to find the optimal stimulation parameters for the four nerves of interest (tibial and peroneal nerves of both feet) using an interactive custom-made GUI. Participants were instructed to describe the intensity of the stimulation they felt on a 0–10 scale, where 0 is not perceived and 10 is very intense, not bearable sensation [40, 41]. The tSTIM was delivered with biphasic rectangular charge-balanced pulses, at a frequency $\:f=50\:Hz$ as previously used in other studies [42–44]. For each nerve, an initial stimulation ramp (biphasic pulses with increasing amplitude (A) and fixed pulse width, $\:PW=300\mu\:s$) was delivered until the participants reported a sensation of 5/10 intensity. If the elicited sensation was perceived as somatotopic (spreading along the nerve and matching the desired foot location), then the electrodes position and stimulation amplitude were saved; otherwise, the electrodes were repositioned until the optimal location was achieved. As a second step, a calibration procedure similar to that reported in [39–41] was conducted to find the perceptual (minimum, 2/10 intensity) and maximum threshold (below pain, 8/10 intensity) for each nerve for each participant. Participants repeated the ramps of fixed A and increasing PW three times. The mean of these PW values across the three repetitions was saved as minimum and maximum PW to modulate the electrical stimulation in the real-time intervention. On the following days, the position of the electrodes, A, and PW of the previous days were used as the starting point of the calibration. Then, when the sensations were not somatotopic or the intensity was not correct, the previous calibration steps were repeated to achieve an optimal, somatotopic sensation.

### Pain assessments

The Neuropathic Pain Symptom Inventory (NPSI) [37] was used as the primary outcome. The NPSI is a standard validated tool for assessing neuropathic pain severity [45]. It is specifically validated to evaluate the different symptoms of neuropathic pain over a 24-h interval. It is scored as the sum of 10 pain descriptor items (rated on a scale from 1 to 10 each).

### Sensory assessments

To measure proprioception, participants underwent the Proprioceptive Displacement Test (Fig. 3A) [39]. During the test, participants sat on a chair and placed their foot on a raised support. While the foot was covered (using a panel) participants were asked to verbally move the pole of a 3D-printed measurement device until it matched the position of their hallux. This was repeated $\:N=10\:$times by repetitively changing the initial position of the foot. The mean error between the reported and correct hallux position was defined as the outcome measure. In other words, the less the displacement is, the more accurate is the body representation of the lower limb. To investigate the impact of the intervention of tactile acuity, the 2PD Test [38] was performed (Fig. 3B). While blindfolded, participants were repetitively touched ($\:N=20$) with either one or two pins at a fixed distance in the plantar and dorsal sides of the foot and asked to determine the correct number of pins [46, 47]. The percentage of success was taken as the outcome measure. Pin distance was individually calibrated before the first session by gradually increasing the pin distance from 10 mm with 5 mm steps. For each distance, participants were touched five times with both pins and were asked to identify the number of pins (one or two). The distance at which participants correctly identified at least two out of five stimuli was selected. The sensory tests were repeated twice in the protocol, on the first and last day of the intervention.

### EEG analysis

Before and after every pain intervention session, participants underwent three minutes of baseline EEG recording. EEG recordings were performed with open eyes and after removal of the VR headset, to avoid mechanical pressure or electromagnetic interference on EEG acquisition. For each recording, EEG signals were band-pass filtered ($\:0.5-42\:Hz$) with a windowed-sinc Hamming Finite Impulse Response (FIR) filter of order 2000. Independent Component Analysis was performed to remove eye and muscular artifacts. The data were then re-referenced to the average of all 24 channels. The analysis was conducted only on the central (Cz, C3, C4, CPz, CP1, CP2) and parietal (Pz, P1, P2) regions, given their key role in cortical pain processing [48]. For each channel, the Power Spectral Density (PSD) distribution was estimated using the Welch Method ($\:window\:size=2s,\:overlap=50\%)$. Mean PSD, absolute and relative spectral power were then computed for five bands of interest: Delta $\:\left(1-4\:Hz\right)$, Theta $\:\left(4-8\:Hz\right)$, Alpha $\:\left(8-13\:Hz\right)$, Beta $\:\left(13-30\:Hz\right)$, and Gamma $\:\left(30-40\:Hz\right)$. Once computed, features were standardized for each participant across all sessions. To investigate the associations between EEG features and reported pain, we conducted a correlation analysis that integrated variations in EEG features with participants’ changes in the NPSI scores.

### Statistical analysis

In each analysis, we selected either parametric or non-parametric tests based on the normality of the data, as determined by the Shapiro tests. For the pain analysis, NPSI decrease among the experimental group was evaluated using clinically significant threshold [49], rather than with statistical tests. Clinical significance was defined as a 30% and 20/100 for NPSI reduction, while substantial clinical significance was defined as 50% reduction i.e., the thresholds considered to define responders endpoints in clinical trials for pain treatment [49–51]. To appropriately compare NPSI decrease between the two intervention groups and account for the longitudinal structure of the data and repeated measures within participants, a linear mixed-effects model was implemented. The model included Group (VR+tSTIM vs. VR-only), Time, and their interaction as fixed effects, and participant-specific random intercepts to account for within-subject variability. For interpretability at individual timepoints, post hoc between-group comparisons of baseline-corrected NPSI changes were conducted using independent t-tests or Mann–Whitney tests, depending on data distribution. For the 2PD and Propioceptive displacement tests, the statistical analysis was carried within groups, using the paired t-test (or the Wilcoxon test, depending on the normality). The correlation between EEG features variation and reported pain variation was carried out with Pearson’s correlation coefficient (or Spearman’s rank correlation coefficient, depending on the normality).

## Results

 Eighteen participants (10 females, 8 males, mean age of $\:66\pm\:12$) with chronic neuropathic pain participated in the study. Of these, thirteen participants developed neuropathic pain as a complication of peripheral diabetic neuropathy; two participants had painful idiopathic neuropathy; two participants had Drug-Induced Peripheral Neuropathy (DIPN) and one participant was diagnosed with Tarsal Tunnel Syndrome (TTS) neuropathy (Table S2). Statistical analyses between the two groups (VR and VR+tSTIM) were performed to ensure comparability in terms of age and NPSI scores (Table S3).

### VR+tSTIM intervention clinically reduces neuropathic pain

The participants' subjective pain analysis was conducted following Dworkin et al. [49] recommendations, hence using two different methods to evaluate the clinical importance of pain improvement. For NPSI, clinically significant thresholds were therefore set at 30% and 20 points reductions from the baseline NPSI (average between WB before and D1 NPSI).

**Fig. 2:**
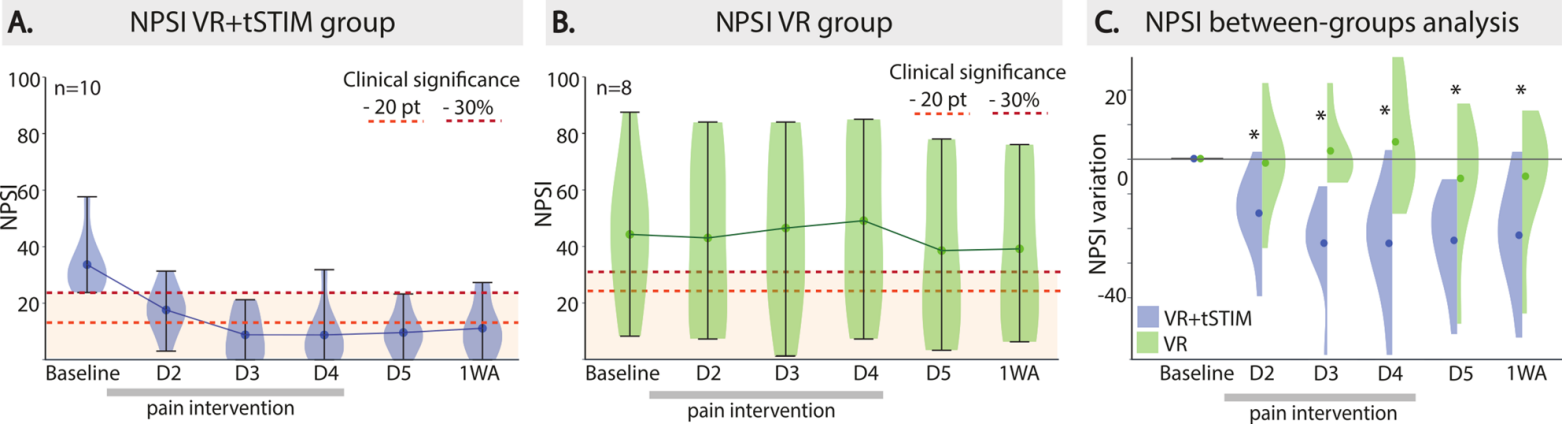
NPSI pain changes. NPSI distribution for VR+tSTIM (**A**) group and VR group (**B**). Each violin plot corresponds to a specific day of measurement, from baseline through days 2 to 5 (the day after therapy completion), and the one week follow up. For each violin, the shaded shape represents the kernel-density estimate of the data distribution, while the dot marks the mean value and the connecting line links the means across timepoints. The red (−30%) and orange (−20pt) lines show clinically significant pain reduction thresholds. **C** Comparisons of NPSI pain reduction from baseline for VR+tSTIM and VR groups as violin plots. T-test or Mann-Whitney tests (depending on normality) were used for statistical analysis. * *p* < 0.05, ** *p* < 0.01, *** *p* < 0.001

Participants in the VR+tSTIM group (*N* = 10) showed a clinically meaningful reduction in NPSI scores based on both clinical thresholds. These improvements emerged by the third day of treatment and persisted through the one week follow up, dropping from baseline ($\:{NPSI}_{baseline}^{VR+tSTIM}=33.3\pm\:9.3$), to day three ($\:{NPSI}_{D3}^{VR+tSTIM}=8.7\pm\:7.7$) and remaining low at one week after the intervention ($\:{NPSI}_{WA}^{VR+tSTIM}=11\pm\:8.7$) (Fig. 2A) (Table S4). At the single participant level, 7 out of 10 individuals exhibited a substantial improvement, with NPSI reductions above 50% [49, 50, 52], from baseline to the one week follow up. One participant showed a moderate reduction above 30% [49, 50, 52]. In contrast, the VR group (*N*=8) did not show clinically meaningful mean changes across days, with no group level reduction relative to baseline (Fig. 2B) (Table S4). Only one participant in this group showed a substantial reduction above 50% at the one week follow-up. Consistent with these observations, the linear mixed-effects model revealed no significant change over time in the VR-only group (β = −1.04, *p* = 0.231), but a significant Group × Time interaction (β = −2.81, *p* = 0.016), indicating divergent pain trajectories and greater reductions in the VR+tSTIM group. Post hoc analyses on baseline-corrected NPSI changes confirmed significantly greater reductions in the VR+tSTIM group compared to VR-only across all intervention days (Fig. 2C, Table S5).

### VR+tSTIM intervention improves tactile acuity and proprioceptive performance

**Fig. 3:**
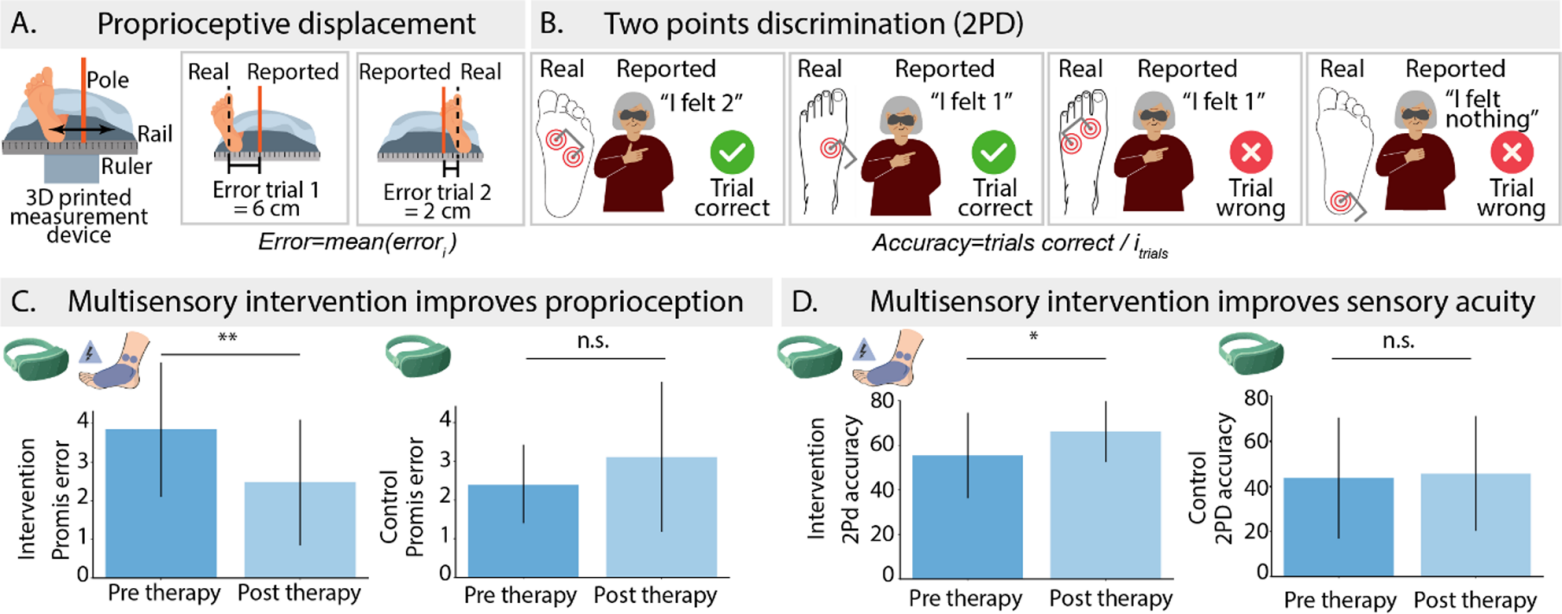
Sensory tests. **A** Proprioceptive displacement protocol. The participant is sitting down with the leg covered. The displacement is defined as the spatial difference between the reported position and the real position of the hallux. **B** Two points discrimination protocol. While blindfolded, participants are repetitively touched ($\:\mathrm{N}=20$) with either one or two pins at a fixed distance and report the perceived number of pins. The percentage of success is taken as the outcome measure. **C** Proprioceptive displacement error for VR+tSTIM and VR group on the first and last day of intervention. **D** Two points discrimination accuracy for the VR+tSTIM and VR group on the first and last day of intervention. Mannwhitney U test, * *p*<0.05, ** *p* < 0.01. n.s.: not significant

Proprioceptive displacement and tactile acuity, defined through the accuracy during two-point discrimination (2PD) test, were measured at the first and last therapy session, for both the VR+tSTIM and the VR group. Proprioceptive displacement, measured as mean error during 10 repetitions, is shown in Fig. 3C. The VR+tSTIM group showed a statistically significant reduction of the proprioceptive error (from 3.89 ± 1.75 to 2.50 ± 1.61, *p* = 0.006). TheVR group showed no improvement (2.40 ± 1.01 to 3.15 ± 1.78 *p* = 0.151). Tactile acuity, measured as the number of correct trials in the 2PD test, is shown in Fig. 3D. Tactile acuity statistically significantly improved from pre to post therapy for the VR+tSTIM group (55.4 ± 19.2 to 66.1 ± 13.7, *p* = 0.012) but not for the VR group (45.6 ± 28.1 to 47.6 ± 26.6, *p* = 0.423).

### Objective EEG digital biomarkers provide evidence for pain reduction

To indentify objective biomarkers supporting the therapeutic efficacy of the intervention, we observed the participants’ neurophysiological EEG changes. Specifically, we computed the difference in EEG features between the first (D1) and the last day (D4) of intervention (Supp. Fig. S1). One participant in the VR+tSTIM group declined EEG recording, resulting in a reduced sample size of *N* = 9 for this analysis. We extracted band-power features representing the EEG information in the frequency domain.

**Fig. 4:**
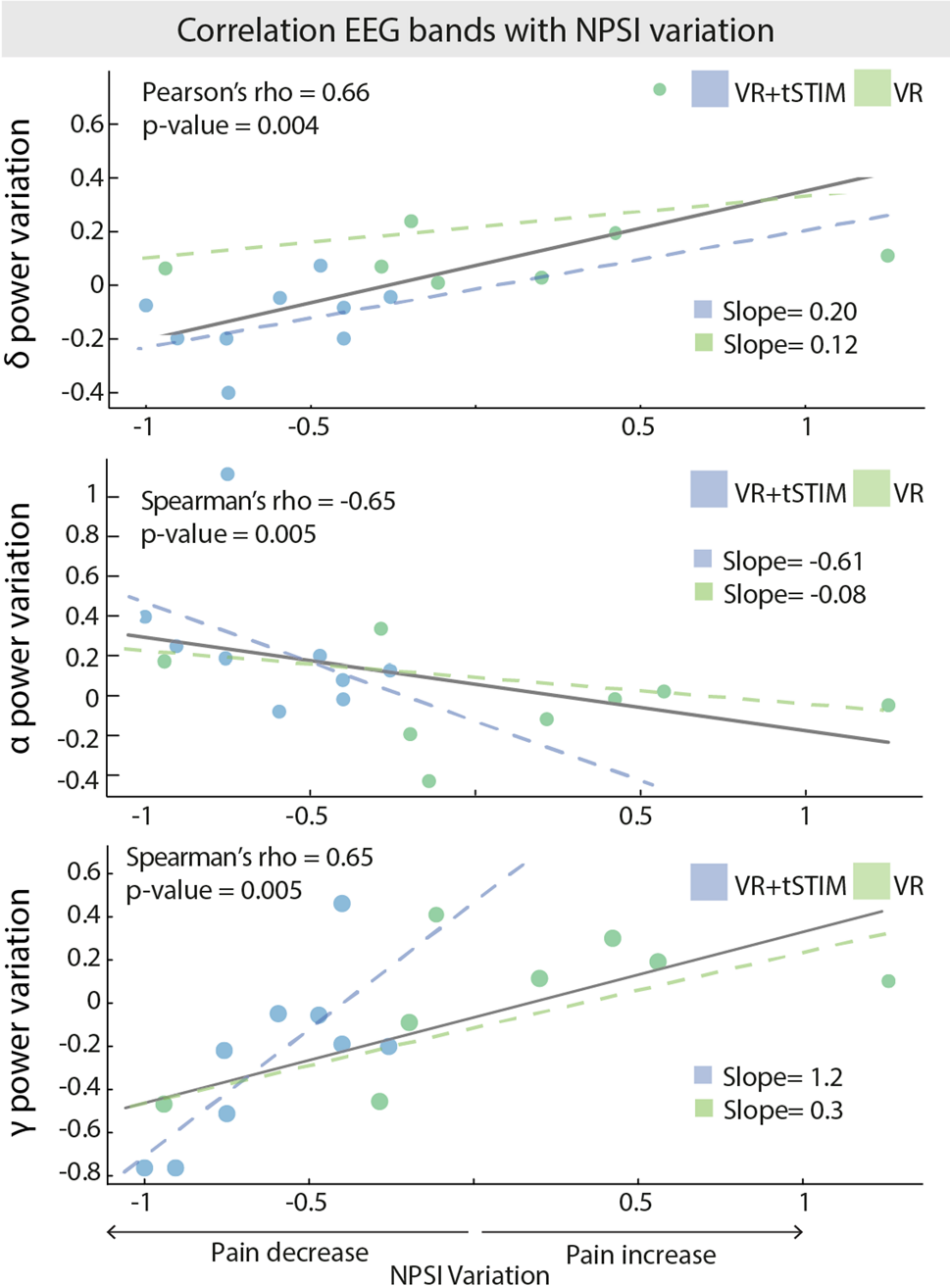
Neurophysiological correlations with pain. Changes of EEG power in the relative delta central region, alpha absolute power in the central region and gamma absolute power in the parietal region shown as a function of NPSI variations, form Day 1 to Day 4 of the intervention, both for the VR+tSTIM group ($\:\mathrm{N}=9)$ and the VR group ($\:\mathrm{N}=8)$. The correlation between the feature variation and the NPSI variation for all participants is shown (grey line). The correlation coefficient and p-value are reported. The correlation of the VR+tSTIM (in blue) and VR (in green) group are also shown. The slopes of the respective linear regressions are reported

 Three representative features of the overall behavior are shown in Fig. 4: δ, α and γ respectively. Change in EEG relative δ power in the central region (from D1 to D4) positively correlates with participants’ NPSI score changes ($\:Spearman\:\rho\:=0.66,\:p=0.004)$ (Fig. 4, Table S6). By performing separate linear regression analysis between the two groups, the slope of line was higher in the VR+tSTIM ($\:{slope}_{VR+tSTIM}=0.20)\:$group compared to the VR group ($\:{slope}_{VR}=0.12$) (Fig. 4A), and the VR+tSTIM showed a lower intercept. The correlation analysis showed a significant negative correlation between the EEG α PSD in central region and participants’ NPSI score changes ($\:Spearman\:\rho\:=-0.65,\:p=0.005)$ (Fig. 4, Table S6). Separated linear regression showed lower slope for the VR+tSTIM ($\:{slope}_{VR+\mathrm{t}\mathrm{S}\mathrm{T}\mathrm{I}\mathrm{M}}=-0.61$) compared to the VR ($\:{slope}_{VR}=-0.08$) (Fig. 4). In the γ band, a significant decrease of EEG absolute parietal γ power showed a significant decrease in NPSI score ($\:Pearson\:\rho\:=0.65,\:p=0.005)$ (Fig. 4C, Table S6), and again a higher slope for the VR+tSTIM group ($\:{slope}_{VR+\mathrm{t}\mathrm{S}\mathrm{T}\mathrm{I}\mathrm{M}}=1.20)\:$compared to the VR group ($\:{slope}_{VR}=0.30)\:$in the separate linear regression analysis.

## Discussion

Demonstrating the clinical efficacy and therapeutic engagement of new therapies is essential for their translation and adoption, yet it remains challenging. Reliable and objective clinical endpoints, including the integration of biomarkers [26, 28], are essential for this to happen. In this work, we propose comprehensive monitoring framework incorporating behavioral, sensory, and neurophysiological assessments to objectively quantify efficacy of an innovative non-pharmacological intervention reducing neuropathic pain.

### Self-reported pain analysis

First, we showed that targeted VR neurostimulation clinically decreases pain as measured through the NPSI scores. Clinical significance is defined as a 30% and 20/100 for NPSI reduction in pain, which are the thresholds considered to define responders endpoints in clinical trials for pain treatment [49–51]. The clinically significant reduction was achieved on the second day of treatment, showing a cumulative effect and persisting up to the follow-up one-week post-intervention.

The comparison with an active comparator is often missing in previous trials [34, 53, 54] and is essential for validating the true therapeutic benefit of the proposed intervention [55]. Studies that only report within-group changes fail to establish whether a treatment is effective, as observed changes might result from factors other than the treatment itself (e.g., contextual biases) [26, 55]. To this end, we demonstrated that the VR-only condition did not yield significant NPSI changes at any time point. Although VR-only can sometimes produce short transient improvements on simpler 1D pain scales like VAS [25], these effects likely reflect attentional or expectancy biases inherent to one-dimensional ratings [56] rather than effective analgesia. Consistent with this, NPSI, which aggregates multiple neuropathic pain subdomains, showed no significant or sustained change, suggesting only a short-term attentional modulation of pain with VR [20, 57].

Additionally, a day-by-day comparison of the two interventions showed that targeted VR neurostimulation produced significantly greater NPSI reductions than VR-only throughout the treatment period and at follow-up. This consistent superiority over an active VR control supports a reliable therapeutic effect of the multimodal intervention, ruling out possible placebo effect of the application of a generic intervention.

### Sensory biomarkers of neuropathic pain

Alongside self-reported pain measures, objective sensory pain readouts were collected. Diminished tactile acuity has been reported in peripheral neuropathies [58], due to lesions of the somatosensory pathways disrupting the transmission of tactile stimuli, and in different chronic pain conditions [59]. Interestingly, tactile acuity, assessed using the 2PD test, showed significant improvement in the VR+tSTIM group following the intervention. A combination of peripheral and central factors can explain this finding [59, 60]. Several studies demonstrated that TENS plays a role in increasing blood circulation [61–64], underscoring its potential impact on nerve damage [65]. Therefore, the provided electrical stimulation (received by the VR+tSTIM group only) may directly influence the integrity of sensory pathways and contribute to improved acuity. At the central level, extensive research showed changes in representational fields in the primary somatosensory cortex associated with alterations in 2PD thresholds [66–70]. Our findings may reflect neuroplastic adaptations in the representational areas of the somatosensory cortex following the VR+tSTIM intervention. These neuroplastic changes likely enhance the brain’s ability to process tactile information more accurately, thus concurrently improving tactile acuity and reducing pain perception. This central perspective could also explain the improvement observed in the proprioceptive displacement test, where participants in the VR+tSTIM group demonstrated an enhanced ability to accurately locate the position of their lower limb. The proposed task tests for participants’ short-term body representation, (i.e. current limb angles and positions) as encoded in their sensorimotor cortex [22]. Evidence indicates that neuropathic pain is associated with structural changes in the somatosensory cortex [66, 71], and generates anomalous body perception (e.g. body parts perceived as “heavy”, “constricted” or “swollen”) [39, 72–74]. In this context, multisensory stimulations (especially visuo-tactile contingencies) have been advocated as effective therapeutic tools for altering the subject’s sense of ‘self’ [21] and directly impacting body representation to alleviate pain [17, 18, 23, 75]. Hence, the observed results are an indicator of the impact of the VR+tSTIM intervention on network dynamics [76] to effectively modulating body representation. The lack of improvement in the VR condition can be explained by the necessity for congruent stimuli from multiple modalities to create a robust and coherent bodily illusion [77]. Since the VR group received only visual stimulation, the same effect on restoring and improving body representation was not observed.

### Neurophysiological indicators of pain

To assess pain changes, self-reported and sensory pain readouts need to be coupled with electrophysiological pain indicators, to comprehensively monitor therapeutic response and disease progression [26, 28]. EEG is a non-invasive and cost-effective method for gathering neurophysiological pain data. While the search for EEG chronic pain biomarkers is extensive [48, 78], most studies are either cross-sectional, comparing chronic pain patients to healthy subjects, or descriptive studies monitoring participants undergoing a single intervention or no intervention [78]. Our study design enables the monitoring of EEG signatures of chronic pain and allows for a correlation analysis between self-reported levels of pain and EEG responses, both in the intervention and control group. We aimed to understand whether the reduction in pain as perceived subjectively by the participants was supported by objective neural changes. In particular, we focused on central and parietal regions because they are closely linked to the sensory discriminative and multisensory integrative dimensions of pain [30, 31, 48]. Central electrodes capture activity over somatosensory areas involved in the processing of nociceptive input, whereas parietal regions are relevant for visuotactile integration and body representation, both of which are especially pertinent to a multisensory intervention combining VR and targeted stimulation. Variations in delta, gamma, and alpha bands aligned with changes in NPSI scores. Gamma and delta power showed larger reductions in participants who experienced stronger pain relief, while alpha power increased proportionally to the analgesic effect. This pattern is consistent with previous work showing that gamma activity is often elevated in chronic pain and is thought to reflect pain related sensory and affective processing, particularly the encoding of nociceptive salience and intensity [48, 79]. Likewise, increased delta activity has been associated with pathological low frequency thalamocortical activity in neuropathic pain states and may reflect abnormal network synchronization linked to persistent pain processing [48, 80]. In contrast, reduced alpha power is commonly reported in chronic pain conditions and is often interpreted as a marker of diminished cortical inhibitory control and persistent hyperexcitability of somatosensory networks [48, 79]. Overall, these correlations suggest that the greater the reduction in pain, the higher the difference in the neural biomarker from pre- to post-therapy. When no reduction in pain was observed (as often for the VR group), the relative neural biomarkers were not showing significant changes. This supports the interpretation that subjective pain relief was accompanied by measurable modulation of cortical oscillatory activity, rather than reflecting only nonspecific or transient effects. Notably, the slope of the fitted linear regression was steeper (in absolute value) for the VR+tSTIM group compared to the VR group. One possible interpretation is that the combined intervention more effectively engaged both bottom-up and top-down mechanisms: tSTIM may have reduced ascending nociceptive input and enhanced sensory gating, whereas VR may have modulated attentional, body related, and multisensory integrative processes. Their combination may therefore have promoted a more substantial normalization of pain related oscillatory activity across these key frequency bands [48, 79].

Overall, our results indicate that multisensory intervention is associated with modulation of neural pathways involved in pain processing, supporting the efficacy of the targeted VR-neurostimulation treatment. These cortical biomarkers are particularly important when participants cannot provide verbal feedback, for example in disabled or paralyzed patients, enabling a communication channel based on objective measurable neurophysiological signals [81].

### Limitations and future perspective

While VR+tSTIM produced clear subjective and objective pain reductions, it remains uncertain whether the sensory improvements are driven by the stimulation itself or arise as a secondary consequence of pain relief. In neuropathic conditions, sensory loss, pain symptoms, and disruptions in body representation are tightly interconnected, making it difficult to determine which mechanism primarily explains the observed improvements. In this context, although the intervention was specifically designed to engage body representation through visuo-tactile synchrony, we did not include a direct measure of embodiment or body ownership in the present study to directly assess this point. However, previous work using a closely related VR-TENS platform has already shown that synchronous visuo-electro-tactile stimulation can effectively modulate embodiment, as demonstrated by both subjective and objective measures [82]. The VR condition in our design helped control for expectations and placebo effects of the therapy, but does not allow us to isolate the contribution of tSTIM alone. To properly disentangle these mechanisms, future studies should include a third condition with tSTIM only. This would clarify whether neurostimulation by itself drives sensory and cortical changes or whether these changes emerge only when stimulation is paired with multisensory VR input. In addition, a broader set of sensory, cortical, and peripheral evaluations will be needed to more fully characterize treatment related changes. Although the present framework captured several relevant dimensions of pain modulation, it does not yet encompass the full range of mechanisms that may be affected by the intervention. High resolution neuroimaging techniques such as fMRI could be used to examine changes in functional connectivity and structural features of the somatosensory cortex following treatment. Complementary neuropathy assessments, including 10 g monofilament testing and nerve conduction studies, would help quantify the severity and progression of sensory impairment and determine whether the intervention produces measurable improvements beyond pain reduction. Finally, incorporating wearable physiological sensors such as wristbands would support the identification of additional pain related neurophysiological markers in real world settings, including autonomic and peripheral signatures not captured by EEG alone. This would help advance portable, data driven tools for monitoring therapeutic responses and mapping pain related patterns over time.

## Conclusions

This study extends our previous exploratory work by providing the first in-depth analysis of the neurophysiological mechanisms underlying the analgesic effects of a multisensory intervention that combines immersive virtual reality with targeted neuromodulation for sensory restoration. By examining concurrent changes in tactile acuity and proprioceptive performance, we show that this integrated approach not only alleviates pain but also promotes reorganization of sensory processing pathways which are known to be affected by neuropathy. Crucially, these behavioral improvements are paralleled by distinct cortical signatures in the EEG, indicating modulation of neural activity in regions involved in pain processing. Together, these findings demonstrate that pain relief emerges from the synergistic interaction between peripheral and central mechanisms, highlighting the potential of multisensory neurostimulation to restore the integrity of sensorimotor and cortical networks disrupted in chronic neuropathic pain.

## Supplementary Information

Supplementary material 1.

## Data Availability

The datasets generated and/or analysed during the current study are not publicly available due restrictions related to participant privacy and institutional policies but all the completely anonymized data, code and materials used in the analysis are available from the corresponding author on reasonable request.

## Abbreviations

2PD: Two-Point Discrimination
DA: Day After (1-day follow-up)
DIPN: Drug-Induced Peripheral Neuropathy
D1-D4: Intervention Days 1–4
EEG: Electroencephalography
FIR: Finite Impulse Response (filter)
GUI: Graphical User Interface
NPSI: Neuropathic Pain Symptom Inventory
PSD: Power Spectral Density
tSTIM: Targeted Neurostimulation
TENS: Transcutaneous Electrical Nerve Stimulation
VR: Virtual Reality
WB: Week Before (baseline timepoint)
WA: Week After (1-week follow-up)
