# Supplementary material for: Sensory and cortical biomarkers unveil pain modulation mechanisms induced by targeted multisensory neurostimulation
Source: J Neuroeng Rehabil. 2026 Apr 29;23:194. doi: 10.1186/s12984-026-01998-5 (PMC13270703; doi:10.1186/s12984-026-01998-5)
Supplement: Supplementary file 1 — Supplementary material 1. [file 12984_2026_1998_MOESM1_ESM.pdf]

Sensory and cortical biomarkers unveil pain modulation mechanisms induced by targeted multisensory neurostimulation

Giuseppe Valerio Aurucci[1,*], Noemi Gozzi[1,*], Andrea Cimolato[2], Markus Wagner[1], Carl Moritz Zipser[3], Stanisa Raspopovic[1,2]

[1] Neuroengineering Laboratory, Department of Health Sciences and Technology, ETH Zürich; Tannenstrasse 1, 8092 Zürich

[2] Center for Medical Physics and Biomedical Engineering, Medical University of Vienna, 1090 Vienna, Austria

[3] Department of Neurology and Neurophysiology and Spinal Cord Injury Center, Balgrist University Hospital, University of Zurich, Zurich, Switzerland

* These authors contributed equally to this work
Corresponding author. Email: nesta.fale@gmail.com

Supplementary materials

Figure S1. Group-averaged post-session power spectral density across intervention days in central and parietal regions of interest.
Table S1. Inclusion and exclusion criteria
Table S2. Participants' characteristics
Table S3. Grouped baseline analysis
Table S4. Patients' NPSI values
Table S5. NPSI variations and p-values
Table S6. EEG features - NPSI correlation analysis
Table S7: Linear mixed-effects models of within-session EEG changes (Post–Pre) across intervention days.

Figure S1. Group-averaged post-session power spectral density across intervention days in central and parietal regions of interest. Group-averaged power spectral density (PSD) curves reconstructed from raw EEG recordings using Welch's method for the central (top row) and parietal (bottom row) regions of interest. PSDs are shown for post-session recordings across intervention days (Day 1–4), separately for the intervention (left column) and control groups (right column).

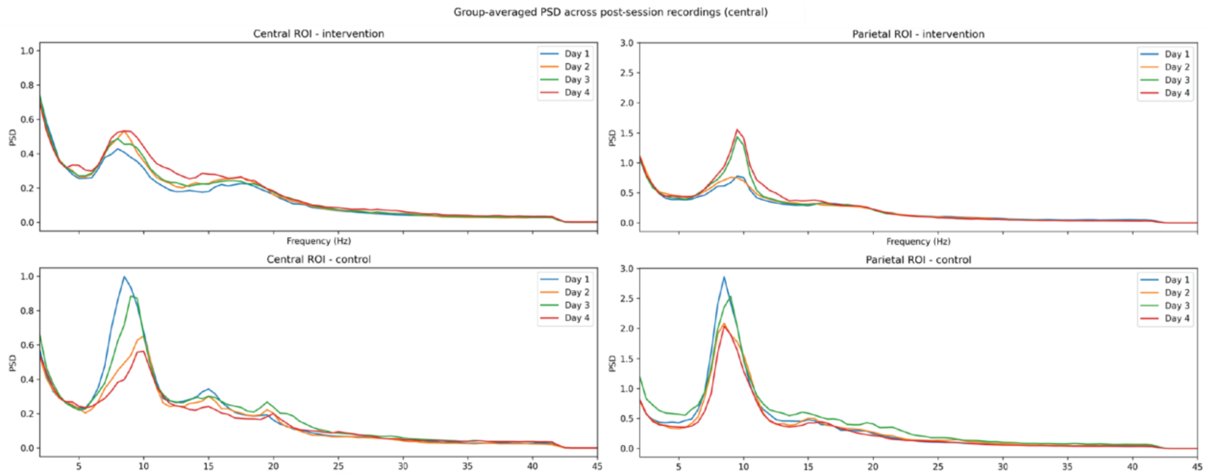

Table S1. Inclusion and exclusion criteria

Inclusion criteria	*Exclusion criteria*
Diagnosed peripheral neuropathy	*Pregnancy*
Pain in lower limbs >= 3 on VAS scale	*Mental Illness*
Pain for more than 3 months	*Pacemaker*
Age 18 - 80	*Epilepsy*

Table S2. Participants' characteristics. DPN = Diabetic Peripheral Neuropathy; DIPN = Drug-Induced peripheral neuropathy, TTS = Tarsal tunnel syndrome. PC01-PC08 are intended as control patients.

	Age (years	Height (cm)	Weight (kg)	BMI (kg/m^2)	Origin of Neuropathy	Time since diagnosis (years)	Experimental condition
**P01**	*77*	*175*	*75*	*25*	*DPN*	*15 years*	*VR+tSTIM*
**P02**	*61*	*180*	*82*	*25*	*DPN*	*11 years*	*VR+tSTIM*
**P03**	*80*	*151*	*59*	*26*	*DPN*	*9 years*	*VR+tSTIM*
**P04**	*70*	*189*	*96*	*27*	*DPN*	*4 years*	*VR+tSTIM*
**P05**	*78*	*180*	*80*	*25*	*DPN*	*16 years*	*VR+tSTIM*
**P06**	*47*	*180*	*68*	*21*	*DIPN*	*2 years*	*VR+tSTIM*
**P07**	*57*	*163*	*50*	*19*	*DIPN*	*2 years*	*VR+tSTIM*
**P08**	*57*	*174*	*61*	*20*	*DPN*	*25 years*	*VR+tSTIM*
**P09**	*79*	*182*	*85*	*25*	*Idiopathic Neuropathy*	*5 years*	*VR+tSTIM*
**P10**	*77*	*168*	*90*	*32*	*DPN*	*30 years*	*VR+tSTIM*
**PC01**	*59*	*190*	*111*	*31*	*DPN*	*13 years*	*VR*
**PC02**	*72*	*190*	*95*	*26*	*DPN*	*23 years*	*VR*
**PC03**	*58*	*170*	*68*	*24*	*TTS*	*2 years*	*VR*
**PC04**	*60*	*165*	*55*	*20*	*Idiopathic Neuropathy*	*1 year*	*VR*
**PC05**	*74*	*159*	*94*	*37*	*DPN*	*40 years*	*VR*
**PC06**	*51*	*178*	*95*	*30*	*DPN*	*26 years*	*VR*
**PC07**	*76*	*176*	*67*	*22*	*DPN*	*25 years*	*VR*
**PC08**	*46*	*158*	*70*	*28*	*DPN*	*8 years*	*VR*

Table S3. Grouped baseline analysis. On the top, Statistical analysis of demographics and clinical data variables (Age, BMI, baseline NPSI, PROMIS and 2-Point Discrimination); on the bottom EEG-related features at baseline (δ-Central relative power, α-Central Power Spectral Density, γ-Parietal absolute power)

	VR+tSTIM (mean +- std)	***VR (mean +- std)***	***pvalue***
Demographics & Clinical Data			
Age	*68 +- 11.8*	*62.3 +- 10.9*	*0.306*
BMI	*24.5 +- 3.8*	*27.2 +- 5.5*	*0.233*
NPSI	*33.3 +- 9.8*	*44.5 +- 24.9*	*0.305*
PROMIS	*3.89 ± 1.75*	*2.40 ± 1.01*	***0.020***
2PD	55.37 ± 19.18	45.61 ± 28.13	0.372
EEG Features (baseline)			
**Central relative power (delta)**	0.111 ± 0.040	0.107 ± 0.038	0.828
**Central PSD (alpha)**	0.044 ± 0.016	0.049 ± 0.017	0.520
**Parietal absolute power (gamma)**	0.836 ± 0.533	0.702 ± 0.288	0.523

Table S4. Patients' NPSI values. *Acronymous: D1, D2, D3, D4: Day 1, Day 2, Day 3, Day 4 of the treatment; 1WB, D5 5, 1WA: One week before, one day and one week after the treatment end respectively.*

	1WB	*D1*	*D2*	*D3*	*D4*	*D5*	*1WA*
P01	*35*	*32*	*26*	*20*	*15*	*13*	*13*
P02	*30*	*30*	*10*	*8*	*10*	*18*	*12*
P03	*41*	*41*	*23*	*13*	*2*	*10*	*8*
P04	*30*	*17*	*11*	*0*	*4*	*9*	*0*
P05	*34*	*39*	*19*	*14*	*2*	*0*	*0*
P06	*28*	*28*	*17*	*3*	*7*	*7*	*13*
P07	*27*	*31*	*31*	*21*	*31.5*	*23*	*25*
P08	*51*	*63*	*17*	*0*	*0*	*6*	*5*
P09	*35*	*15*	*17*	*0*	*12*	*9*	*27*
P10	*29*	*29*	*3*	*8*	*3*	*0*	*7*
PC01	*28*	*49*	*32*	*34*	*37*	*35*	*35*
PC02	*51*	*51*	*55*	*53*	*52*	*3*	*6*
PC03	*27*	*8*	*13*	*19*	*31*	*18*	*14*
PC04	*83*	*92*	*82*	*84*	*79*	*74*	*76*
PC05	*74*	*50*	*84*	*84*	*84*	*78*	*76*
PC06	*66*	*45*	*59*	*65*	*85*	*64*	*62*
PC07	*11*	*5*	*11*	*1*	*7*	*6*	*10*
PC08	*33*	*33*	*7*	*31*	*17*	*29*	*33*

Table S5. NPSI variations and p-values. *Baseline is the average between week before and Day 1. Acronymous: D1, D2, D3, D4: Day 1, Day 2, Day 3, Day 4 of the treatment; 1DA, 1WA: One day and one week after the treatment end respectively.*

	$\Delta NPSI_{VR+tSTIM}(mean \pm std)$	$\Delta NPSI_{VR}(mean \pm std)$	***p-values***
Baseline	-	-	-
D2	*-15.85 ± 10.93*	*-1.25 ± 12.66*	*0.025*
D3	*-24.55 ± 12.19*	*2.25 ± 8.4*	*<0.001*
D4	*-24.6 ± 15.24*	*4.88 ± 14.49*	*0.001*
1DA	*-23.75 ± 12.66*	*-5.75 ± 17.97*	*0.032*
1WA	*-22.25 ± 14.83*	*-5.13 ± 16.64*	*0.026*

Table S6. EEG features - NPSI correlation analysis. Upward arrows indicate a positive correlation between feature value and NPSI variation from day 1 to day 4. Downward arrows indicate the opposite. Depending on normality, Pearson or Spearman correlation were used (↑↑: p <= 1.00e-02; ↑: p <= 0.05; -= non-significant).

	PSD POW δ	***REL POW δ***	***PSD δ***	***PSD POW ϑ***	***REL. POW ϑ***	***PSD ϑ***	***PSD POW α***	***REL POW α***	***PSD α***	***PSD POW β***	***REL. POW er β***	***PSD β***	***PSD POW γ***	***REL POW γ***	***PSD γ***
central	-	↑↑	↑↑	-	-	-	-	↓↓	↓↓	-	-	-	↑	↑↑	↑↑
parietal	-	-	-	-	-	-	↓	-	-	-	-	-	↑↑	↑↑	↑↑

Table S7: Linear mixed-effects models of within-session EEG changes (Post–Pre) across intervention days. Fixed effects include Group, Day, and their interaction (Group × Day), with random intercepts for subjects.

Feature	***Term***	***Estimate***	***SE***	***z***	***p_value***
central_psdalpha	*Intercept*	*-0.0069*	*0.0025*	*-2.7723*	***0.0056***
central_psdalpha	*Group[T.intervention]*	*0.0082*	*0.0034*	*2.3693*	***0.0178***
central_psdalpha	*Day[T.2]*	*-0.0014*	*0.0031*	*-0.4594*	*0.6459*
central_psdalpha	*Day[T.3]*	*0.0011*	*0.0031*	*0.3587*	*0.7198*
central_psdalpha	*Day[T.4]*	*-0.0004*	*0.0031*	*-0.1229*	*0.9022*
central_psdalpha	*Group[T.intervention]:Day[T.2]*	*-0.002*	*0.0042*	*-0.4696*	*0.6386*
central_psdalpha	*Group[T.intervention]:Day[T.3]*	*-0.0085*	*0.0042*	*-2.0127*	***0.0441***
central_psdalpha	*Group[T.intervention]:Day[T.4]*	*-0.004*	*0.0043*	*-0.9279*	*0.3535*
central_psdalpha	*Group Var*	*0.3272*	*0.2435*	*1.3435*	*0.1791*
central_rel. power delta	*Intercept*	*0.017*	*0.0061*	*2.8054*	***0.005***
central_rel. power delta	*Group[T.intervention]*	*-0.0207*	*0.0084*	*-2.4733*	***0.0134***
central_rel. power delta	*Day[T.2]*	*0.0049*	*0.0085*	*0.5752*	*0.5651*
central_rel. power delta	*Day[T.3]*	*-0.0095*	*0.0085*	*-1.1146*	*0.265*
central_rel. power delta	*Day[T.4]*	*-0.0123*	*0.0085*	*-1.4533*	*0.1462*
central_rel. power delta	*Group[T.intervention]:Day[T.2]*	*0.002*	*0.0117*	*0.1703*	*0.8648*
central_rel. power delta	*Group[T.intervention]:Day[T.3]*	*0.0195*	*0.0117*	*1.6693*	*0.095*
central_rel. power delta	*Group[T.intervention]:Day[T.4]*	*0.0228*	*0.0118*	*1.9251*	*0.0542*
central_rel. power delta	*Group Var*	*0.0271*	*0.1169*	*0.2318*	*0.8167*
parietal_abs. power gamma	*Intercept*	*-0.0703*	*0.166*	*-0.4233*	*0.672*
parietal_abs. power gamma	*Group[T.intervention]*	*-0.0663*	*0.2282*	*-0.2904*	*0.7715*
parietal_abs. power gamma	*Day[T.2]*	*0.1024*	*0.2348*	*0.4363*	*0.6626*
parietal_abs. power gamma	*Day[T.3]*	*0.5052*	*0.2348*	*2.1516*	***0.0314***
parietal_abs. power gamma	*Day[T.4]*	*0.4545*	*0.2348*	*1.9358*	*0.0529*
parietal_abs. power gamma	*Group[T.intervention]:Day[T.2]*	*0.0706*	*0.3227*	*0.2189*	*0.8267*
parietal_abs. power gamma	*Group[T.intervention]:Day[T.3]*	*-0.4381*	*0.3227*	*-1.3577*	*0.1746*
parietal_abs. power gamma	*Group[T.intervention]:Day[T.4]*	*-0.3661*	*0.3274*	*-1.1183*	*0.2634*
parietal_abs. power gamma	*Group Var*	*0*	*0.1391*	*0*	*1*
